# Stability of ascorbic acid in human aqueous humor

**DOI:** 10.1186/s13104-024-06829-1

**Published:** 2024-06-26

**Authors:** Sakae Ito, Mayumi Nagata, Yuka Takino, Akihito Ishigami, Tadashi Senoo

**Affiliations:** 1 Department of Ophthalmology, Dokkyo Medical University, 880 Kitakobayashi, Shimotsuga-gun, Tochigi, 321-0293 Japan; 2 Molecular Regulation of Aging, Tokyo Metropolitan Institute for Geriatrics and Gerontology, 35-2 Sakae-cho, Itabashi-ku, Tokyo, 173-0015 Japan

**Keywords:** Aqueous humor, Ascorbic acid, Antioxidant, High performance liquid chromatography

## Abstract

**Objective:**

The stability of ascorbic acid (AA) in the human aqueous humor (AqH) remains unclear. This study aimed to investigate the stability of AqH AA under varying conditions (27, 4, − 20, and − 80 °C) without acidification.

**Results:**

Rapid AA degradation occurred at 27 °C. At 4 °C, a significant 12.2% degradation was observed after 24 h. Storage at − 20 °C resulted in a notable 37.5% degradation after 28 days, whereas storage at − 80 °C resulted in 10.7% degradation after 28 days. Unacidified AqH samples recorded early decomposition at 27 °C and 4 °C. In conclusion, it is recommended to conduct measurements within 28 days for samples stored at − 80 °C.

## Introduction

Ascorbic acid (AA) concentration in the human aqueous humor (AqH) exceeds 20-fold that of peripheral venous blood [1–3]. This elevated AA concentration, a potent antioxidant, is believed to shield the eye from oxidative stress. Moreover, AA is considered a radical scavenger in the eye because it has a strong reduced action. In some diseases, such as cataracts, glaucoma, and exfoliation syndrome, AA concentration in the AqH has been measured, and its influence on the disease has been considered [1–3]. Conversely, dehydroascorbic acid (DHA) is known to be toxic to the lens and has been linked to the formation of cataracts [4, 5]. Although DHA is preferentially transported into the lens and corneal endothelium, DHA conversion to AA is rapid [6, 7]. However, AA and DHA are highly unstable molecules, with measurement outcomes significantly affected by the type of stabilizer, storage time, and storage temperature. In blood samples, decomposition begins immediately after storage at temperatures of 4 °C and 25 °C [8, 9]. Moreover, stable long-term storage without acidification by trichloroacetic acid or metaphosphoric acid, even at low temperatures, is impractical [9, 10]. In clinical settings, immediate acidification or storage at extremely low temperatures, such as − 80 °C, is difficult due to transport to the laboratory. To the best of our knowledge, no previous studies have examined the stability of AA and DHA in aqueous humor. This study aimed to investigate the difference in AA and DHA stability depending on the storage temperature of AqH, determining optimal storage conditions without acidification.

## Materials and methods

This study included 24 eyes of 24 patients who underwent small incision cataract surgery at Dokkyo Medical Hospital, Japan. A minimum sample volume of 150 μL AqH was aseptically collected at the initiation of surgery following administration of topical anesthesia. The collected sample was divided equally at 30 μL each into five microtubes and promptly stored under four conditions: 27, 4, − 20, and − 80 °C. Under 27 and 4 °C conditions, AA concentration was measured at collection and at 3, 6, 12, and 24 h. Under − 20 and − 80 °C conditions, AA concentration was estimated immediately at collection and at 7, 14, 28, and 56 days.

### Measurement of AA and DHA

AA and DHA were analyzed using a recently published high performance liquid chromatography (HPLC)-electrochemical detection method for precision [10]. These compounds were detected using a Waters 2695 separations module coupled with a Waters 2465 electrochemical detector (Nihon Waters, Tokyo, Japan). Samples were reduced with 35 mM tris (2-carboxyethyl) phosphine hydrochloride (Tokyo Chemical Industry, Tokyo, Japan) for 2 h on ice. Following reduction, the reaction mixture underwent analysis for total AA (AA + DHA) using an HPLC-electrochemical detection method. Separation was performed on an Atlantis dC18 5-μm column (4.6 × 150 mm) with an Atlantis dC18 5-μm guard column (4.6 × 20 mm) (Nihon Waters). The mobile phase comprised 50 mM phosphate buffer (pH 2.8), 540 μM EDTA, and 2% methanol. The flow rate was maintained at 1.3 mL/min, and electrical signals were recorded using an electrochemical detector featuring a glassy carbon electrode at + 0.6 V. DHA content was calculated by subtraction of AA from total AA.

### Statistical analysis

Results are presented as mean ± standard error. Normality was tested using the Shapiro–Wilk test for each time, and normality was not rejected. One-way analysis of variance was conducted using Statistical Package for Social Sciences version 29.0 (IBM Corp., Armonk, NY, USA).

## Results

**Fig. 1:**
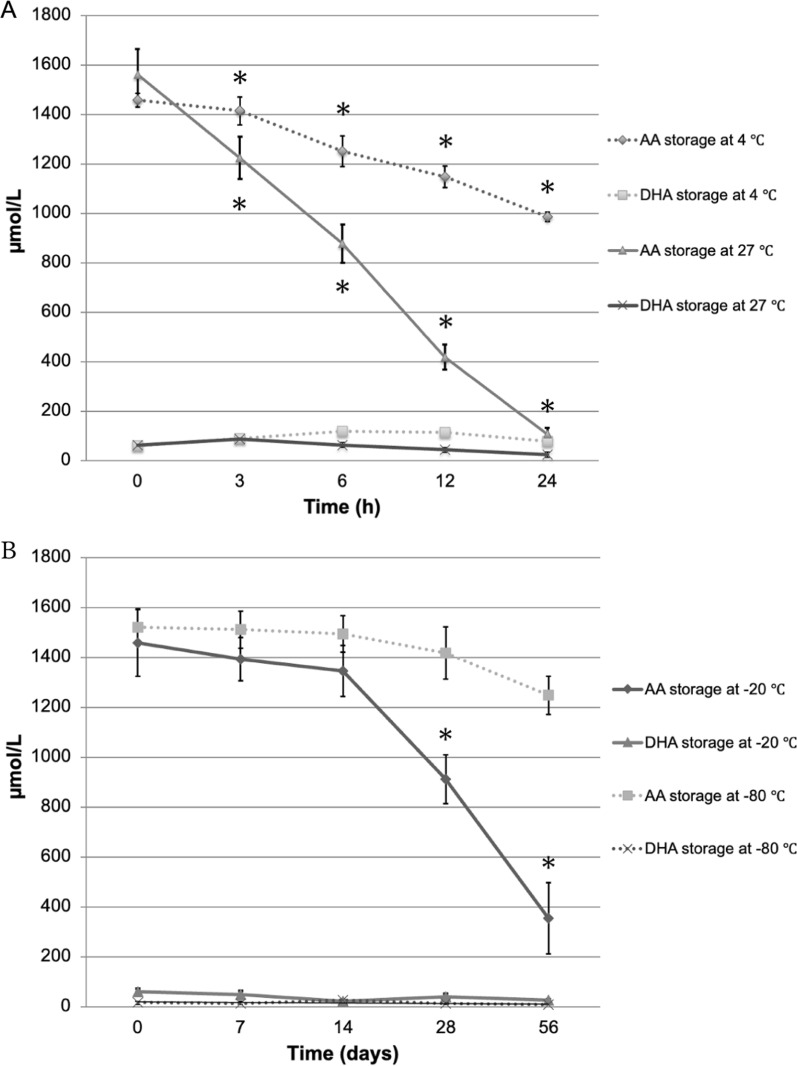
Effect of storage temperature and time on AA and DHA. **A** Variation of AqH AA and DHA in samples stored at 27 °C and 4 °C. **B** Variation of AqH AA and DHA in samples stored at − 20 °C and − 80 °C. Values are presented as mean values at time intervals in hours. *p < 0.05 compared to baseline samples. Data series bars represent standard error. *AqH* aqueous humor, *AA* ascorbic acid, *DHA* dehydroascorbic acid

The mean AA concentrations in AqH samples stored at 27 °C after collection and measured after 3, 6, 12, and 24 h were 1562 ± 103, 1224 ± 86, 877 ± 78, 418 ± 51, and 106 ± 26 μmol/L, respectively. The mean DHA concentrations in the same samples were 62 ± 12, 87 ± 18, 63 ± 14, 43 ± 7, and 24 ± 3 μmol/L, respectively (Table 1; Fig. 1A). The mean AA concentrations in AqH samples stored at 4 °C after collection and at 3, 6, 12, and 24 h were 1457 ± 28, 1415 ± 56, 1251 ± 61, 1147 ± 44, and 985 ± 19 μmol/L, respectively. The mean DHA concentrations in AqH samples stored at 4 °C after collection and at 3, 6, 12, and 24 h were 60 ± 25, 88 ± 15, 118 ± 25, 114 ± 23, and 77 ± 14 μmol/L, respectively (Table 1; Fig. 1A). A rapid degradation of AA was observed in the AqH stored at 27 °C. AqH stored at 4 °C showed a significant 14.1% (P = 0.003) degradation after collection for 12 h.

**Table 1:** Variation of AqH AA and DHA in samples stored at 27 °C and 4 °C

Temperature		Time of storage				
		Baseline	3 h	6 h	12 h	24 h
27 °C	AA	1562 ± 103	1244 ± 86	877 ± 78	418 ± 51	106 ± 26
	p		0.050	< 0.001	< 0.001	< 0.001
	DHA	62 ± 12	87 ± 18	63 ± 14	43 ± 7	24 ± 3
	p		0.646	1.00	0.853	0.263
4 °C	AA	1457 ± 28	1415 ± 56	1251 ± 61	1147 ± 44	985 ± 19
	p		0.978	0.074	0.003	< 0.001
	DHA	60 ± 25	88 ± 15	118 ± 25	114 ± 23	77 ± 14
	p		0.935	0.492	0.549	0.990

Results are expressed as mean ± standard error. Means were compared using One-way ANOVA

*AA* ascorbic acid, *DHA* dehydroascorbic acid

The mean AA concentrations of AqH samples stored at − 20 °C after collection and at 7, 14, 28, and 56 days were 1458 ± 134, 1393 ± 87, 1346 ± 103, 911 ± 98, and 354 ± 143 μmol/L, respectively. The mean DHA concentrations in the same samples were 59 ± 12, 48 ± 16, 22 ± 6, 39 ± 13, and 26 ± 4 μmol/L, respectively (Table 2; Fig. 1B). The mean AA concentrations of AqH samples stored at − 80 °C after collection and at 7, 14, 28, and 56 days were 1522 ± 72, 1511 ± 74, 1494 ± 74, 1418 ± 104, and 1248 ± 76 μmol/L, respectively. The mean DHA concentrations of the same samples were 16 ± 2, 13 ± 2, 27 ± 6, 12 ± 4, and 7 ± 3 μmol/L, respectively (Table 2, Fig. 1B). The collected AqH samples stored for 28 days at − 20 °C showed significant degradation at 37.5% (P = 0.038), whereas those stored at − 80 °C showed only 10.7% (P = 0.917) degradation. No statistically significant differences were observed in DHA concentrations under all conditions.

**Table 2:** Variation of AqH AA and DHA in samples stored at − 20 °C and − 80 °C

Temperature		Time of storage				
		Baseline	7 days	14 days	28 days	56 days
− 20 °C	AA	1458 ± 134	1393 ± 87	1346 ± 103	911 ± 98	354 ± 143
	p		0.996	0.970	0.038	< 0.001
	DHA	59 ± 12	48 ± 16	22 ± 6	39 ± 13	26 ± 4
	p		0.965	0.247	0.786	0.339
− 80 °C	AA	1522 ± 72	1511 ± 74	1494 ± 74	1418 ± 104	1,248 ± 76
	p		1.000	0.999	0.917	0.216
	DHA	16 ± 2	13 ± 2	27 ± 6	12 ± 4	7 ± 3
	p		0.988	0.324	0.982	0.617

Results are expressed as mean ± standard error. Means were compared using One-way ANOVA

*AA* ascorbic acid, *DHA* dehydroascorbic acid

## Discussion

Similar to blood samples [8, 9], AqH samples rapidly decomposed at 27 and 4 °C. Without acidification, blood AA decomposes to undetectable levels after 3 months when stored at − 20 °C [8]. In this study, non-acidified AqH samples stored at − 20 °C exhibited a statistically significant decrease in AA levels from day 14 onward. A previous report suggested that non-acidified blood samples can be stored for up to 20 days at − 70 °C without degradation [8]. Our findings confirm that non-acidified AqH samples can be stored for up to 28 days at − 80 °C without degradation.

The oxygen partial pressure in AqH is approximately 10 to 20 mmHg [11], lower than that in venous blood. It was believed that the oxidation reaction AA may be more difficult to proceed in AqH than in blood. However, because the composition of various substances, such as metal ions, glucose, and other antioxidants, is different between AqH and blood [12, 13], it is difficult to predict differences in the stability of AA. Considering that AA decomposition via oxidation reactions may differ between AqH and blood, no major differences in AA stability were observed in AqH compared to blood samples.

In conclusion, it is recommended that AqH samples stored at − 80 °C be measured within 28 days; furthermore, temperatures of 4 and 27 °C are best avoided.

## Limitations

This study had some limitations. As other analysis methods were not tested, the possibility of different results cannot be ruled out. Moreover, several other antioxidant proteins in human AqH were not measured [14]. Other antioxidant proteins in AqH may influence the oxidation of reduced AA and degradation of DHA. Finally, DHA levels did not significantly change under any of the conditions. Lykkesfeldt discussed an in vivo equilibrium between AA and DHA and recommended the use of internal standards in DHA measurement [15]. This study did not use internal standards in DHA measurement, which may have resulted in low accuracy. Additionally, no direct and sensitive methods currently exist to detect DHA, and the subtraction methods have their limitations [16].

## Data Availability

The data have not been placed in any online data storage. They can be provided on request to the authors.

## Abbreviations

AqH: Aqueous humor
AA: Ascorbic acid
DHA: Dehydroascorbic acid
HPLC: High performance liquid chromatography
